# The Association between eGFR and the Aldosterone-to-Renin Ratio and Its Effect on Screening for Primary Aldosteronism

**DOI:** 10.1155/2020/2639813

**Published:** 2020-02-07

**Authors:** Jing Xu, Yumei Yang, Yan Ling, Zhiqiang Lu, Xin Gao, Xiaomu Li, Xiaoying Li

**Affiliations:** Department of Endocrinology and Metabolism, Zhongshan Hospital, Fudan University, Shanghai 200032, China

## Abstract

**Objectives:**

Long-term exposure to excessive aldosterone secretion from the adrenal gland may cause renal damage in patients with primary aldosteronism (PA). The aldosterone-to-renin ratio (ARR) may be significantly affected by renal function, especially in patients with renal damage related to long-term PA. The objective of this study was to investigate the association between the estimated glomerular filtration rate (eGFR) and ARR as well as its effect on screening for PA.

**Methods:**

This study was performed in Zhongshan Hospital, Fudan University, China. 803 patients with hypertension were consecutively recruited from 2012 to 2015. All participants underwent routine biochemical measurements, including plasma renin activity (PRA) and plasma aldosterone concentration (PAC). In all patients with a PAC higher than 10 ng/dl, a saline perfusion test was conducted, and a CT scan or adrenal venous sampling was also performed if needed. Receiver operating characteristic (ROC) analysis was conducted in all eGFR < 90 and eGFR ≥ 90 groups separately to determine the optimal cut-off values of ARR.

**Results:**

The optimal cut-off point for PA was an ARR of 40 ng/dl per ng/ml.h in the whole population, 52 ng/dl per ng/ml.h in subjects with an eGFR higher than 90 ml/min/1.73 m^2^, and 18 ng/dl per ng/ml.h in subjects with an eGFR lower than 90 ml/min/1.73 m^2^. Patients with an eGFR higher than 90 ml/min/1.73 m^2^ had significantly lower PRA and higher ARR levels than patients with an eGFR lower than 90 ml/min/1.73 m^2^ (*P* < 0.05).

**Conclusions:**

Unsuppressed renin and lower ARR levels were associated with decreased eGFR in patients with primary aldosteronism. Diagnostic criteria of ARR by stratified eGFR may be an optimal strategy for the screening of primary aldosteronism.

## 1. Introduction

Primary aldosteronism (PA) is one of the most common causes of secondary hypertension in adults, with prevalence ranging from 3.2 to 20% in different populations [1]. The aldosterone-to-renin ratio (ARR) is recommended as the screening test for PA [2]. Various cut-off values of ARR were used in different studies or clinical centers, with the majority ranging from 25 to 100 ng/dl per ng/ml/h [3]. Many studies have examined the characteristics of ARR and demonstrated that ARR measurement is a useful screening test for the evaluation of suspected PA [4–6]. Numerous factors are known to affect the ARR, including posture, salt intake, hypokalemia, antihypertension or other medications, and age [7–9]. Thus, before the measurement of ARR, it is recommended that a common posture be used, salt intake be balanced, hypokalemia be corrected, and confounding medications be stopped [2]. However, some factors, including age and gender, cannot be fully avoided and may affect ARR. Therefore, considering the effects of age on the value of ARR, recent studies have calculated the age-specific cut-off value of ARR, which may improve the sensitivity or specificity in the screening for PA [10, 11]. Other confounding factors that influence ARR in addition to age or gender must be fully considered in the screening for PA.

Renal function and glomerular filtration rate are closely associated with the regulatory role of renin synthesis and secretion [12] and the renin-angiotensin aldosterone system [13]. Renin levels have been shown to be significantly suppressed in response to excess aldosterone secretion from the adrenal gland, which caused sodium retention and blood pressure (BP) rises in PA patients. However, several PA cases with unsuppressed plasma renin activity (PRA) and “normal” range ARR values have been reported [14], and these cases have a relatively long history of PA and chronic kidney disease (CKD). Therefore, the effects of PA-associated renal impairment on the renin, RAAS, and value of ARR need to be further evaluated. Catena et al. first reported the correlation between PRA and renal function in PA cases, and their findings suggest that PRA levels may still be induced by decreased glomerular filtration rate in cases with renal damage caused by prolonged PA [15]. Therefore, the changes of PRA and ARR levels in patients with long-term PA-related renal damage may be a unique confounding factor in the screening for PA.

Considering the use of ARR in screening for PA, the effects of renal function on the PRA and ARR values need to be further clarified. In the present study, we will use estimated glomerular filtration rate (eGFR) as the major indicator of kidney function. The eGFR-associated change in ARR values may lead to an increased risk of a false diagnosis of PA in a hypertensive patient if the criteria are determined using patients with normal renal function. On the other hand, a lower threshold could result in too many false positives in the normal renal function group. An ideal optimized cut-off for initial outpatient screening for PA would have high sensitivity, be stable and simple to use, and be cost-effective.

This study was aimed to investigate the association between eGFR and PRA and ARR values in PA patients and to determine the optimal eGFR-dependent screening thresholds.

## 2. Materials and Methods

### 2.1. Study Population and Diagnostic Protocol

**Figure 1:**
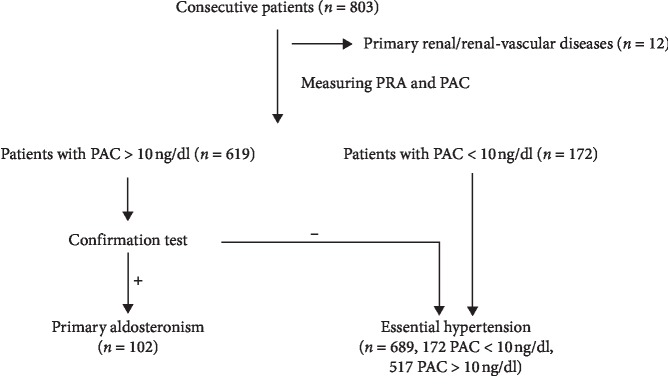
Flow diagram of the study.

Patients with hypertension were consecutively enrolled from the Hypertension Clinic of Zhongshan Hospital, Fudan University, Shanghai, China, from 2012 to 2015. All of the hypertension patients met the 1999 WHO/ISH criteria [16]. Written informed consent was obtained from all of the participants, and the study was approved by the ethics committee of Zhongshan Hospital, Fudan University, China. Patients with a history of diagnosed secondary hypertension (such as endocrinology hypertension and renal hypertension) or known endocrinology diseases (such as hyperthyroidism and pituitary adenoma) were excluded. As shown in Figure 1, a total of 803 consecutive patients were included in this study, and patients with renal/renal-vascular diseases were excluded. In addition, 12 patients with renal vascular disease (diagnosed by color Doppler ultrasound) were further excluded from our study. All the hypertensive patients were required to withdraw agents that affect the ARR, including spironolactone, eplerenone, amiloride, or potassium-wasting diuretics for at least 4 weeks and beta-blockers, angiotensin-converting enzyme inhibitors (ACEI), or angiotensin receptor blockers (ARB) for at least 2 weeks. Hypertensive patients were stabilized with calcium-channel blockers and/or alpha-blockers if needed. Non-dihydropyridine calcium-channel blocker was used in hypertensive patients because it had less impact on RAAS system.

### 2.2. Clinical and Biochemical Measurements

Clinical data including age, gender, and duration of hypertension, the status of BP control, numbers, and dosage of all medications and history of other chronic diseases, including primary kidney disease, were obtained from the clinical documents of the Hypertension Clinic. According to the routine protocol, the results of a complete physical examination including height, weight, waist circumference, body mass index (BMI), and BP were recorded. After 5 min of rest in a sitting position, systolic blood pressure (SBP) and diastolic blood pressure (DBP) were measured in participants by a nurse using a mercury sphygmomanometer adapted for arm size.

BP was measured twice with a 5 min interval, and the mean was used for the analysis. Resistant hypertension was defined in our study as the following criteria: on each of three measurements obtained on different days, with hypertension (BP > 140/90) resistant to three conventional antihypertensive drugs (including a diuretic), or controlled BP (>140/90) on four or more antihypertensive drugs. The general laboratory tests included measurements of serum sodium, potassium, blood urea nitrogen (BUN), and serum creatinine (Scr) levels. eGFR was calculated using the abbreviated Modification of Diet in Renal Disease Study Group (MDRD) equation. Patients were divided into two groups according to the median value of eGFR.

Venous blood was sampled in the morning two hours after waking, after 5–10-minute rest in a sitting position. Plasma aldosterone concentration (PAC) and plasma renin activity (PRA) were measured by radioimmunoassay. PRA was measured by indirect assay involving the generation of angiotensin I (Beifang institute, Beijing, China). The lower limit of detection for PRA in EH and PA subgroup is 0.02 ng/ml/h. Saline perfusion test (SPT) was conducted in all patients with a PAC higher than 10 ng/dl (shown in Figure 1) [17]. A PAC level after saline perfusion higher than 10 ng/dl was considered to be a positive result. In patients with a positive confirmation test, CT scan images were obtained using standard contiguous, 3 mm thick sections/slices with intravenous contrast administration with a GE Mx 8000 Multi-Slice Spiral CT scanner during breath holding. Adrenal venous sampling (AVS) was conducted in cases with unilateral lesions if surgery was desired. All the confirmed unilateral PA patients underwent unilateral adrenalectomy, and the remaining patients received spironolactone treatment.

### 2.3. Conclusive Diagnosis

Among patients with positive saline perfusion tests, a diagnosis of PA was further considered. For subtyping, the diagnosis of aldosterone-producing adenoma required fulfillment of all of the following criteria: unilateral excessive aldosterone secretion confirmed by AVS and evidence of a unilateral adrenocortical nodule on CT scan, pathologically confirmed adenoma after surgery, and complete biochemical success during the postadrenalectomy follow-up. Patients for whom surgery was not indicated received spironolactone treatment. Idiopathic hyperaldosteronism (IHA) was diagnosed in patients with bilateral hyperplasia of the adrenal gland by CT scan, normokalemia and the normalization of BP (140/90 mmHg without the aid of antihypertensive agents) after spirolactone treatment, or significant improvement of hypertension (decrease in mean BP by 20% and fewer antihypertensive agents taken to control BP after spirolactone treatment at follow-up).

### 2.4. Statistical Analysis

Normally continuous variables were expressed as the mean ± standard deviation (SD); the non-normally-distributed variables were expressed as the median and interquartile range. Non-normally-distributed values were log-transformed before analysis. Comparisons between the groups were performed with Student's *t*-test, one-way ANOVA, or *χ*^2^ test for continuous or categorical variables. Spearmen correlations were performed to investigate the possible correlation between ARR and eGFR in PA patients. Sensitivity and 1 − specificity for different cut-offs of the ARR were plotted in receiver operating characteristic (ROC) curves. The optimal cut-off point was assessed using Youden's J statistic. The analyses were performed using SPSS software for Windows, version 13.0 (SPSS, Chicago, IL). *P* value <0.05 was considered to be statistically significant.

## 3. Results

### 3.1. Flow Diagram of the Study

As shown in Figure 1, a total of 803 consecutive patients were included in this study; 12 patients with renal/renal-vascular diseases were further excluded. 619 whose PAC was higher than 10 ng/dl received a saline perfusion test. Finally, 102 patients were diagnosed with PA, and 689 patients were diagnosed with essential hypertension (EH). In this study, 158 (125 and 33 in EH and PA group, respectively) out of 791 patients reached the criteria of resistant hypertension. According to our original data, we found that, among 37 patients with unilateral lesions, 25 patients underwent AVS in this study. Among the 25 patients undergoing AVS, 16 patients were finally diagnosed with unilateral APA via pathology.

### 3.2. The Correlation of eGFR with ARR in EH and PA patients

The general clinical information of both PA and EH patients is shown in Table 1. The age and gender distributions were similar between these two groups. PA patients had lower serum potassium and higher systolic and diastolic blood pressures than the EH patients (*P* < 0.05). In each group, all subjects were divided into two subgroups according to the median eGFR (90 ml/min/1.73 m^2^ in EH or PA separately, Table 1). In the EH group, patients with higher eGFR were younger and had lower SBP and PRA levels (*P* < 0.05) and higher borderline ARR levels (*P*=0.06) than patients with lower eGFR. In the PA group, patients with higher GFR were younger, were younger at onset of hypertension, had lower SBP, DBP, and PRA levels, and had significantly higher ARR levels than patients with lower eGFR (*P* < 0.05). The differences in ARR levels remained significant after adjustment for age, gender, and hypertension duration (*P* < 0.05). In this study, correlations were performed and our results indicated that eGFR significantly correlated with ARR levels in PA cases (shown in Supplementary [Supplementary-material supplementary-material-1]).

The differences in ARR levels remained significant after adjustment for age, gender, and hypertension duration (*P* < 0.05).

### 3.3. Test Characteristics and Screening Accuracy of eGFR-specific Cut-Off Points for ARR

**Figure 2:**
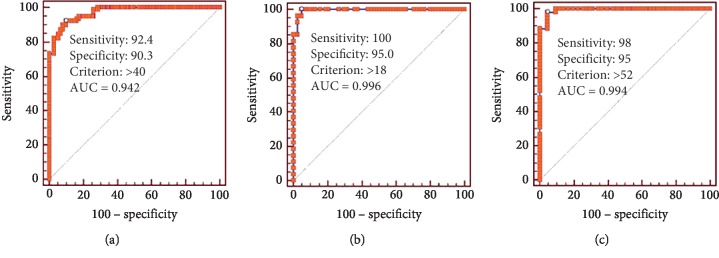
Receiver operating characteristic curves according to eGFR. Evaluation of ARR accuracy for the screening for PA using ROC curve analysis in the full study cohort (a), in those with eGFR < 90 ml/min/1.73 m^2^  (b), and in those with eGFR ≥ 90 ml/min/1.73 m^2^ (c). ARR: aldosterone-to-renin ratio; eGFR: estimated glomerular filtration rate; PA: primary aldosteronism; ROC: receiver operating characteristic curves.

ROC analysis was conducted in all eGFR <90 and eGFR ≥90 groups separately. ROC curves were shown in Figure 2 and Table 2. The area under the curve (AUC) was 0.942 (95% CI 0.922 to 0.978, *P* < 0.001) in all (Figure 2(a)), 0.996 (95% CI 0.979–1.000, *P* < 0.001) in eGFR < 90 (Figure 2(b)), and 0.994 (95% CI 0.980 to 0.999, *P* < 0.001) in eGFR ≥ 90 patients (Figure 2(c)). Optimal cut-off values of ARR which corresponded to Youden's index were also yielded and were 40 ng/dl per ng/ml/h in all patients (the sensitivity and specificity were 56.5% and 93.0%), 18 ng/dl per ng/ml/h in the eGFR < 90 group (the sensitivity and specificity were 100.0% and 95.3%), and 52 ng/dl per ng/ml/h in the eGFR ≥ 90 group (the sensitivity and specificity were 98.1% and 95.4%). As shown in Table 2, when using 40 or 52 ng/dl per ng/ml/h as the cut-off point for a diagnosis of PA in the eGFR <90 group, the sensitivity decreased to 85.2%, whereas when using 18 or 40 ng/dl per ng/ml/h as the cut-off for the diagnosis of PA in eGFR ≥90 group, the specificity decreased to 90.7%. According to our comparison, 8 (8/49, 16.3%) PA patients with eGFR <90 will be misdiagnosed when not using the optimal cut-off point, compared with conventional cut-off value in our study. Lower cut-off point used in CDK patients was meaningful to avoid missed diagnosis.

**Figure 3:**
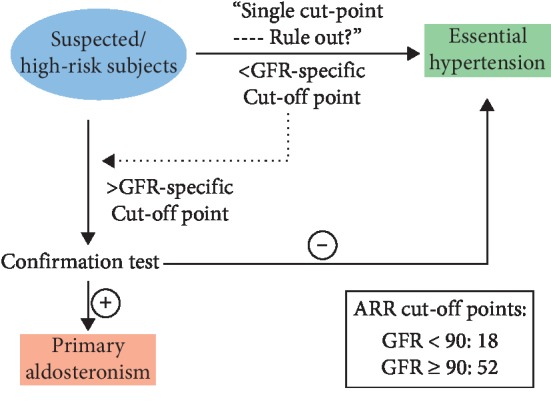
Recommended approach for the use of ARR in the screening for PA. The protocol was interpreted as follows. Firstly, we enrolled subjects in accordance with the inclusion criteria and exclusion criteria. Then confirmation tests were conducted in every hypertension case with PAC higher than 10 ng/dl to fully ensure no PA cases were missed, regardless of the ARR value. Finally, PA was carefully diagnosed in cases with positive confirmation test results and clinical outcomes.

Based on the above analysis, Figure 3 shows the recommended approach for the use of eGFR levels and ARR to screen for PA.

## 4. Discussion

In the present study, we accessed the performance characteristics of ARR in screening for PA. We found a significant positive correlation between ARR and eGFR in PA patients, and we included an analysis of PA patients with different eGFR levels. Our data make it possible to explore an eGFR-dependent strategy for improving the accuracy of screening for PA. These results may also inform other physicians or researchers who must select appropriate cut-off points for diagnosing PA, especially in patients with decreased renal function.

It has been well established and widely accepted to use ARR in the screening for PA [3]. However, it is very important to stress that using ARR for the detection of PA must be confirmed by further testing for a final diagnosis. Our modified ARR may be used as a cost-effective screen for detection of cases needing further confirmation tests. There are a number of potential pitfalls in using ARR for detection PA which clinicians should be aware of. ARR levels are significantly altered by several antihypertension medications [2], which should be stopped before the measurements of ARR. Potassium status and sodium loading may also need to be adjusted in advance. However, some other factors may be unavoidable before screening. Advanced age has been demonstrated to affect the ARR value, and an age-dependent cut-off point may improve the accuracy of using ARR in the screening for PA [10, 11, 18]. Similarly, the effects of gender such as a female-specific cut-off point in ARR have also been reported [19, 20]. In the present study, we recommended that renal function should also be considered in the screening for PA using ARR.

Our results indicated that eGFR significantly correlated with ARR levels in PA cases, and this correlation was mainly due to elevated renin levels in cases with decreased renal function. Previously, it has been reported that primary kidney diseases and renal failure patients had higher ARR levels [21]. Renal failure with increased levels of creatinine may lead to false positives when using ARR in the screening for PA [2]. However, in patients without a history of previous kidney disease, the long-term PA-caused renal damage is different from primary kidney diseases, which may alter the ARR levels in a distinct pattern. The effects of renal function and eGFR on renin or ARR levels in PA patients are still controversial due to the differences of case selection and population [15, 22–24]. To be consistent with our findings, escape of renin from suppression by excess aldosterone secretion was also reported to be associated with evidence of more severe renal damage in patients with PA in an Italian population [15]. In addition, higher ARR levels have been reported to be an independent predictor of eGFR in pretreated PA patients [24], and PRA levels were also reported to be correlated with kidney impairment in the TAIPAI study.

Our results indicated that decreased eGFR was associated with increased PRA levels and decreased ARR levels in PA cases. Aldosterone is autonomously synthesized and secreted independent of the renin-angiotensin system (RAS) in PA cases. The variation of ARR may be significantly affected by renin levels among these patients. Renin is synthesized by juxtaglomerular epithelioid cells in the medial layer of renal afferent arterioles. Renal function and glomerular filtration rate are the major factors influencing renin synthesis and secretion in normal and pathophysiological situations. Under prolonged hypertension and high aldosterone levels, decreased eGFR may also stimulate the synthesis and secretion of renin in PA patients. Accordingly, unsuppressed PRA may contribute to lower ARR values in PA cases with renal damage. Our results showed that decreased eGFR was strongly associated with substantially increased PRA and earlier onset of hypertension in PA cases, which might reflect the natural history in patients with long-term PA.

Considering the significant key findings on the correlation between ARR and eGFR, it is evident that eGFR-specific diagnostic criteria can be used to optimize the sensitivity and specificity of screening for PA. The proposed decision limits of patients with an eGFR below 90 ml/min/1.73 m^2^ (18 ng/dl per ng/dl/h) and patients with an eGFR above 90 ml/min/1.73 m^2^ (52 ng/dl per ng/dl/h) will improve the accuracy of using ARR. It is strongly recommended to use a decreased cut-off point (18 ng/dl per ng/dl/h) instead of using the single cut-off point (40 ng/dl per ng/dl/h) in patients with eGFR below 90 ml/min/1.73 m^2^; otherwise, the sensitivity would dramatically decrease to 85.2%, which would lead to the misdiagnosis of 8 (8/49, 16.3%) PA patients whose eGFR was below 90 ml/min/1.73 m^2^.

The protocol used in our study tried to ensure an accurate diagnosis of PA. Confirmation tests were conducted in every hypertension case with PAC higher than 10 ng/dl to fully ensure no PA cases were missed, regardless of the ARR value. PA was carefully diagnosed in cases with positive confirmation test results and clinical outcomes. The strict diagnosis criteria used here strongly support the diagnosis among PA patients.

There were still several limitations in our study: (1) confirmation tests for PA were not conducted in all cases; (2) we only used eGFR to evaluate renal function; it would be more accurate enough if we included more parameters; (3) this study was a monocentric study and we only enrolled patients from one medical institution. At present, we are not yet able to perform any external validity due to lack of participants elsewhere. We would explore the internal validity and external validity in the future to make the results of our study be more broadly applicable; (4) our study was retrospective; therefore, we could not identify cause-and-effect relationship of PA and renal damage. Follow-up data remained to be included to further investigate the relationships of plasma renin and aldosterone levels with pre- and posttreatment renal function; (5) the current literature does not identify a gold-standard confirmatory test for PA, so we have also taken the operative diagnosis, effective surgery, and medication together into account to obtain a final diagnosis of PA.

## 5. Conclusions

ARR must now be regarded as an initial test, with further diagnostic confirmation tests for PA in patients with hypertension. Using eGFR-dependent decision limits will optimize the diagnostic accuracy of using ARR; decision values of 18 ng/dl per ng/dl/h (eGFR < 90 ml/min/1.73 m^2^) and 52 ng/dl per ng/dl/h (eGFR ≥ 90 ml/min/1.73 m^2^) can be recommended as optimized cut-off points.

## Figures and Tables

**Table 1:** General characteristics in essential hypertension and primary aldosteronism patients.

	EH			PA		
	ALL	GFR < 90	GFR ≥ 90	ALL	GFR < 90	GFR ≥ 90
	(*n* = 689)	(*n* = 344)	(*n* = 345)	(*n* = 102)	(*n* = 49)	(*n* = 53)
Age (year)	51.2 ± 1.0	62.7 ± 1.2	**42.9** **±** **1.3**^*∗*^	54.1 ± 0.9	57.3 ± 1.3	**51.6** **±** **1.3**^**§**^
Gender (M/F)	150/152	61/67	79/85	68/70	38/24	30/46
BMI (kg/m^2^)	27.5 ± 7.2	27.0 ± 7.5	27.9 ± 7.8	26.8 ± 4.9	27.0 ± 4.5	26.5 ± 4.7
Year of HT (year)	4 (1–9)	4 (1–11)	3 (1–8)	5 (2–10)	6 (3–11)	**4 (2–8)** ^**§**^
eGFR (ml/min/h)	89.5 ± 18.6	64.6 ± 20.0	108.4 ± 19.9	91.0 ± 19.6	74.7 ± 1 8.7	104.4 ± 18.1
SBP (mmHg)	132.2 ± 19.1	134.6 ± 18.7	**130.3** ± **18.5**^*∗*^	141.4 ± 18.6	143.0 ± 20.1	**139.3** **±** **20.5**^**§**^
DBP (mmHg)	79.9 ± 10.7	79.8 ± 11.0	80.0 ± 10.8	85.7 ± 12.0	87.3 ± 11.4	**83.7** **±** **12.5**^**§**^
Serum K^+^ (mmol/L)	4.0 ± 2.1	4.2 ± 2.1	4.0 ± 2.2	3.3 ± 2.1	3.4 ± 1.9	3.3 ± 1.8
BUN (mmol/L)	5.8 ± 1.5	6.8 ± 1.8	5.0 ± 1.4	5.5 ± 1.4	6.5 ± 1.8	4.9 ± 1.3
Scr (umol/L)	75.8 ± 15.3	92.4 ± 17.0	64.5 ± 13.0	72.9 ± 16.9	90.6 ± 18.2	60.9 ± 11.7
FBG (mmol/L)	5.3 ± 2.1	5.4 ± 2.2	5.3 ± 2.0	5.5 ± 2.1	5.6 ± 2.0	5.4 ± 2.2
ARR^+^ (ng/dl per ng/ml/h)	11.0 (6.4–20.0)	10.7 (5.9–14.0)	**13.7** ^*∗#*^ **(7.7–30.0)**	98.8 (59.6–207.4)	68.7 (40.3–135.0)	**122.6** ^**§$**^ **(79.7–262.5)**
**PRA** ^**+**^ **(ng/ml/h)**	**1.0 (0.6**–**2.0)**	1.3 (0.9–2.4)	0.8 (0.4–1.9)	0.2 (0.1–0.3)	0.3 (0.1–0.5)	**0.2** ^**§**^ **(0.1-0.2)**
PAC^+^ (ng/dl)	1.351 (0.846–1.529)	1.333 (0.806–1.556)	1.359 (0.946–1.508)	1.814 (1.721–2.239)	1.804 (1721–2.230)	1.856 (1.725-2.254)

Continuous data are expressed as means ± standard deviation. ^+^Variables were log-transformed before statistical analysis; numbers in the table were back-transformed as median (interquartile range). Significant difference between GFR ≥90 and GFR <90 groups in EH (^*∗*^) or PA (^§^). Significant difference between GFR ≥ 90 and GFR < 90 groups in EH (^#^) or PA (^$^) after adjustment for age, gender, and hypertension duration (*P* < 0.05). ARR, aldosterone-to-renin ratio; eGFR, estimated glomerular filtration rate; EH, essential hypertension; PA, primary aldosteronism; BMI, body mass index; BUN, blood urea nitrogen; Scr, serum creatinine.

**Table 2:** Test characteristics of ARR to screen for PA according to stratified GFR.

eGFR	Optimal cut-off	Sensitivity (95% CI)	Specificity (95% CI)	Positive LR (95% CI)	Negative LR (95% CI)
ALL	18	100.0 (95.4–100.0)	71.38 (67.6–75.0)	3.5 (3.1–4.0)	—
	**40**	**92.4 (84.2–97.2)**	**90.3 (87.7–92.6)**	**9.6 (7.4–12.3)**	**0.1 (0.0-0.1)**
	52	82.3 (72.1–90.0)	94.7 (92.6–97.3)	15.5 (10.9–22.0)	0.2 (0.1–0.3)
<90	**18**	**100.0 (87.2–100.0)**	**95.3 (92.0–97.6)**	**21.3 (12.3–37.1)**	**—**
	40	85.2 (66.3–95.8)	100.0 (98.6–100.0)	—	0.2 (0.1–0.4)
	52	85.2 (66.3–95.8)	100.0 (98.6–100.0)	—	0.2 (0.1–0.4)
≥90	18	100.0 (93.2–100.0)	90.7 (87.2–93.6)	10.8 (7.8–15.0)	—
	40	100.0 (93.2–100.0)	90.7 (87.2–93.6)	10.8 (7.8–15.0)	—
	**52**	**98.1 (89.7–100.0)**	**95.4 (92.6–97.3)**	**21.2 (13.1–34.2)**	**0.0 (0.0-0.1)**

ARR, aldosterone-to-renin ratio; eGFR, estimated glomerular filtration rate; PA, primary aldosteronism; LR, likelihood ratios; CI, confidence interval.

## Data Availability

The data used to support the findings of this study are available from the corresponding author upon request.

## Supplementary Materials

Supplementary Materials Supplementary Table 1: correlations between ARR and eGFR in EH and PA patients. Supplementary Table 2: comparison of diagnostic performance with different cut-off points in PA patients according to stratified GFR.
