# Supplementary material for: The Association between eGFR and the Aldosterone-to-Renin Ratio and Its Effect on Screening for Primary Aldosteronism
Source: Int J Endocrinol. 2020 Feb 7;2020:2639813. doi: 10.1155/2020/2639813 (PMC7029269; doi:10.1155/2020/2639813)
Supplement: Supplementary Materials — Supplementary Table 1: correlations between ARR and eGFR in EH and PA patients. Supplementary Table 2: comparison of diagnostic performance with different cut-off points in PA patients according to stratified GFR. [file 2639813.f1.docx]

Supplementary

TABLE 1 Correlations between ARR and eGFR in EH and PA patients.

| eGFR |  | ALL | EH | PA |
| --- | --- | --- | --- | --- |
| PRA | r | -0.332 | 0.325 | -0.408 |
|  | p | 0.001***** | 0.001***** | 0.001***** |
| PAC | r | 0.083 | 0.123 | 0.044 |
|  | P | 0.03***** | 0.05 | 0.703 |
| ARR | r | 0.167 | 0.390 | 0.375 |
|  | P | 0.001***** | 0.001***** | 0.001***** |

* indicates statistically significant.

Abbreviations: PRA, plasma renin activity; PAC, plasma aldosterone concentration; ARR, aldosterone-to-renin ratio

TABLE 2 Comparison of diagnostic performance with different cutoff points in PA patients according to stratified GFR

|  | PA | | |
| --- | --- | --- | --- |
|  | ＜40 | | ＞40 |
| **eGFR＜90** |  |  |  |
| ＜18 | 0 | | 0 |
| ≥18 | **8** | | 41 |
| **eGFR>90** |  |  |  |
| ＜52 | 1 | | 0 |
| ≥52 | 0 | | 52 |

According to our comparison, 8 (8/49, 16.3%) PA patients with eGFR< 90 will be misdiagnosed if not using the optimal cutoff point, compared with conventional cutoff value in our study.
